# ASSOCIATION BETWEEN SOCIAL POLICY, STIGMA, SOCIAL ACCEPTANCE, AND MENTAL HEALTH AMONG OLDER MEN WHO HAVE SEX WITH MEN

**DOI:** 10.1093/geroni/igae098.3197

**Published:** 2024-12-31

**Authors:** Alex Siu Wing Chan, Mark Lachmann, Hok Bun Ku, Elsie Yan

**Affiliations:** The Hong Kong Polytechnic University, Hong Kong, Hong Kong, China (People’s Republic); Mount Sinai Hospital, Toronto, Ontario, Canada; The Hong Kong Polytechnic University, Hong Kong, Hong Kong, China (People’s Republic); The Hong Kong Polytechnic University, Hong Kong, Hong Kong, China (People’s Republic)

## Abstract

**Background:**

Older MSM (Men who have Sex with Men) often face discrimination and marginalization due to ageism and homophobia, impacting their psychological well-being. This study investigates the influence of social factors on the psychological well-being of older MSM, considering variations in social policy and cultural contexts across China, Hong Kong, and Taiwan.

**Methods:**

A comparative approach was utilized, gathering data (N=453) from older MSM in the aforementioned regions. Measures assessing social policy, stigma, social acceptance, and psychological well-being were employed. Control variables, such as age, education level, and income, were included to mitigate potential influences. The data were analyzed using descriptive statistics, ANOVA, and regression analysis.

**Results:**

The findings demonstrate significant negative effects of stigmatization across all three regions. F-statistics indicate the regression models’ overall significance: China (F = 69.583, p < 0.001), Hong Kong (F = 51.971, p < 0.001), and Taiwan (F = 57.725, p < 0.001). This highlights stigmatization’s strong influence on dependent variables in each region. Additionally, high adjusted R-squared values suggest substantial explained variance: China (Adj R2 = 0.632), Hong Kong (Adj R2 = 0.514), and Taiwan (Adj R2 = 0.565), emphasizing the model’s ability to account for variability in dependent variables.

**Conclusion:**

The study underscores the need for targeted interventions and policy changes to combat stigma and promote social acceptance, thereby enhancing the psychological well-being of this vulnerable population.

